# Research Progress in Small-Molecule Detection Using Aptamer-Based SERS Techniques

**DOI:** 10.3390/bios15010029

**Published:** 2025-01-08

**Authors:** Li Zheng, Qingdan Ye, Mengmeng Wang, Fan Sun, Qiang Chen, Xiaoping Yu, Yufeng Wang, Pei Liang

**Affiliations:** 1 College of Optical and Electronic Technology, China Jiliang University, Hangzhou 310018, China; s23040809016@cjlu.edu.cn (L.Z.); p23040854145@cjlu.edu.cn (Q.Y.); p23040854112@cjlu.edu.cn (M.W.); 2 Key Laboratory of Microbiological Metrology, Measurement & Bio-Product Quality Security, State Administration for Market Regulation, Zhejiang Provincial Key Laboratory of Biometrology and Inspection & Quarantine, College of Life Sciences, China Jiliang University, Hangzhou 310018, China; s23090710042@cjlu.edu.cn (F.S.); yxp@cjlu.edu.cn (X.Y.); 3 College of Metrology and Measurement Engineering, China Jiliang University, Hangzhou 310018, China; chenqiang_cjlu@cjlu.edu.cn

**Keywords:** small molecule, aptamer, SERS, sensors

## Abstract

Nucleic acid aptamers are single-stranded oligonucleotides that are selected through exponential enrichment (SELEX) technology from synthetic DNA/RNA libraries. These aptamers can specifically recognize and bind to target molecules, serving as specific recognition elements. Surface-enhanced Raman scattering (SERS) spectroscopy is an ultra-sensitive, non-destructive analytical technique that can rapidly acquire the “fingerprint information” of the measured molecules. It has been widely applied in qualitative and trace analysis across various fields, including food safety, environmental monitoring, and biomedical applications. Small molecules, such as toxins, antibiotics, and pesticides, have significant biological effects and are harmful to both human health and the environment. In this paper, we mainly introduced the application and the research progress of SERS detection with aptamers (aptamer-based SERS techniques) in the field of small-molecule detection, particularly in the analysis of pesticide (animal) residues, antibiotics, and toxins. And the progress and prospect of combining the two methods in detection were reviewed.

## 1. Introduction

Small molecules generally refer to organic compounds with a molecular weight of less than 1000 Daltons, including a variety of natural and synthetic compounds such as toxins, antibiotics, hormones, dyes, pesticides, and more [1] (Lyashchenko and Cremers, 2021). With the widespread use of small molecules, the sensitive and accurate detection of small-molecule targets has significant implications for clinical medical diagnostics, environmental monitoring, food safety control, and other fields. In the field of small-molecule-residue detection, methods based on high-performance liquid chromatography (HPLC) [2], chromatography-mass spectrometry (LC-MS) [3], chemiluminescent immunoassay (CLIA) [4], and others, can achieve high selectivity and quantitative detection. However, these methods have drawbacks, such as being time-consuming, expensive, complex to operate, and requiring specific instruments and trained personnel. In recent years, the combination of aptamer and Surface-enhanced Raman spectroscopy (SERS) technologies has become an effective tool for high sensitivity and accurate small-molecule detection. This approach has opened new areas for small-molecule detection technology, ranging from environmental and food pollutant detection to diagnostic and therapeutic applications. SERS technology offers advantages such as high sensitivity, unique fingerprint identification, and nondestructive analysis. Furthermore, advancements in biotechnology and computational techniques have improved aptamer screening methods, enhancing the selectivity of aptamer recognition and the precision and efficiency of capturing target analytes. By integrating aptamers with SERS technology, a sensor based on small-molecule aptamers can combine the high specificity of aptamers with the high sensitivity of SERS, thereby effectively expanding their potential in practical applications. As highly specific recognition elements, aptamers will contribute to human health and social progress in areas such as biomedical, environmental monitoring, and food safety.

This review highlights the latest developments and applications of aptamer and SERS methods in the field of small-molecule detection. Following a systematic introduction to the basics of both techniques, the application of aptamer-based SERS technology in small-molecule detection will be discussed.

## 2. Introduction to Aptamers

### 2.1. Definition of Aptamer

**Figure 1:**
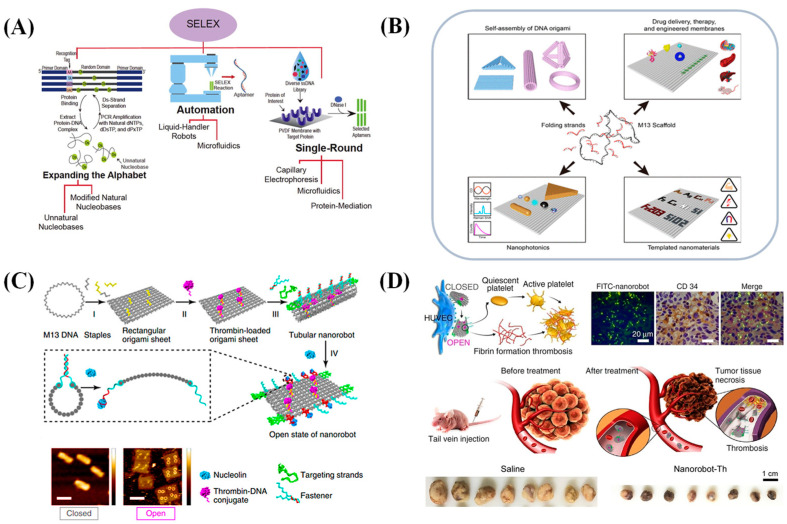
(**A**) Schematic diagram of the principle of SELEX [13]. (**B**) Schematic diagram of DNA origami nanotechnology [14]. (**C**) Schematic diagram of the DNA nanorobot based on DNA origami technology [15]. (**D**) The principle of the smart drug delivery system [15].

The aptamer is a highly structured oligonucleotide sequence (DNA or RNA) [5] obtained through the Systematic Evolution of Ligands by Exponential Enrichment (SELEX) technique [6]. SELEX is a recognized and essential method for aptamer screening. To date, a large number of aptamers have been isolated from artificial RNA or DNA libraries using the SELEX method. The process generally follows these steps (as shown in Figure 1A): (1) A random oligonucleotide library with a sequence capacity of approximately 10^13^–10^15^ is designed, referred to as the initial library. The target (such as a protein or small molecule) is then immobilized on a solid support (e.g., magnetic beads or microplates), and the oligonucleotide library is incubated with it. (2) The nucleic acid-target complexes are separated via amplification and purification, and a sub-library is prepared for the first round of screening. (3) The sub-library is cycled with the target through steps (1) and (2). Through multiple rounds of binding, washing, and amplification, aptamers with high affinity and specificity are enriched, resulting in an affinity-enriched library. (4) The affinity-enriched library is sequenced, and the sequences with the best binding performance to the target are selected as aptamers.

The selected aptamers can bind to a specific target with high affinity and selectivity and are often referred to as “chemical antibodies”. The term ‘aptamer’ was first coined in 1990 by A.D. Ellington and JW Szostak, who conducted an experiment showing that RNA molecules were able to bind synthetic organic dyes present in a randomized pool of RNA [7,8], and another experiment was conducted by Craig Tuerk and Larry Gold. Gold performed another experiment in which new RNA sequences were found in a synthetic RNA pool that functioned similarly to natural RNA sequences that bind to T4 DNA polymerase [6]. The aptamers fold into three-dimensional structures and are able to bind to their targets with high specificity and selectivity through structural recognition, emphasizing the specific interaction relationship between nucleic acid aptamers and their target molecules [9,10].

In recent years, aptamers have become promising biorecognition elements in sensor construction. Many aptamers targeting small molecules have been screened and used as recognition elements in various biosensors. Aptamers show a broad prospect in scientific research and clinical applications due to their advantages of high specificity, affinity, nonimmunogenicity, chemical modification, and rapid mass production. They are used as recognition probes, and they are also applied in DNA nanotechnology, drug delivery, bioimaging, sensor construction, etc., which promote the development of biomedicine. On the basis of the law of complementary pairing between bases, aptamers are able to self-assemble into diverse secondary structures, such as rings, hairpins, stem structures, etc. The spatial conformation of these spontaneous folding structures is the key for achieving high-affinity interactions with ligands. It is based on this unique self-folding ability of nucleic acids that scientists have developed DNA origami, an advanced DNA nanotechnology. DNA origami, a cutting-edge DNA nanotechnology, was first proposed by Rothemund [11], where a description of the technology details its underlying principles and application potential. The core concept of the technology is to self-assemble a long-stranded DNA (scaffold) with multiple short-stranded DNA (staples) into a predetermined nanostructure in solution by specific DNA sequence design, as shown in Figure 1B. This innovative approach to DNA nanostructure design has greatly contributed to the advancement of nanotechnology.

Li et al. [12] constructed a DNA nanorobot capable of responding to molecular triggers and playing a therapeutic role in tumors based on DNA origami technology, which was designed as a smart drug delivery system capable of responding to molecular triggers and specifically transporting payloads (e.g., thrombin) to the tumor site, as shown in Figure 1C,D. This nanorobot’s outer surface is coated with DNA aptamers that specifically recognize nuclear proteins, which are primarily found on the endothelial cells associated with tumors, which allows the nanorobot to precisely target tumor sites. Additionally, these aptamers serve as molecular triggers. Upon binding to the nuclear proteins, they initiate a structural change within the nanorobot, leading to the release of thrombin. The thrombin then activates the coagulation process at the tumor location, causing the formation of blood clots within the tumor’s blood vessels. This process results in tumor cell death and slows down tumor growth. The experimental results showed that the DNA nanorobot successfully delivered thrombin specifically to tumor-associated blood vessels in a hormonal mouse model, produced significant antitumor effects, and was immunologically inert, indicating its good biocompatibility and application prospects.

### 2.2. Classification of Small-Molecule Aptamers

**Figure 2:**
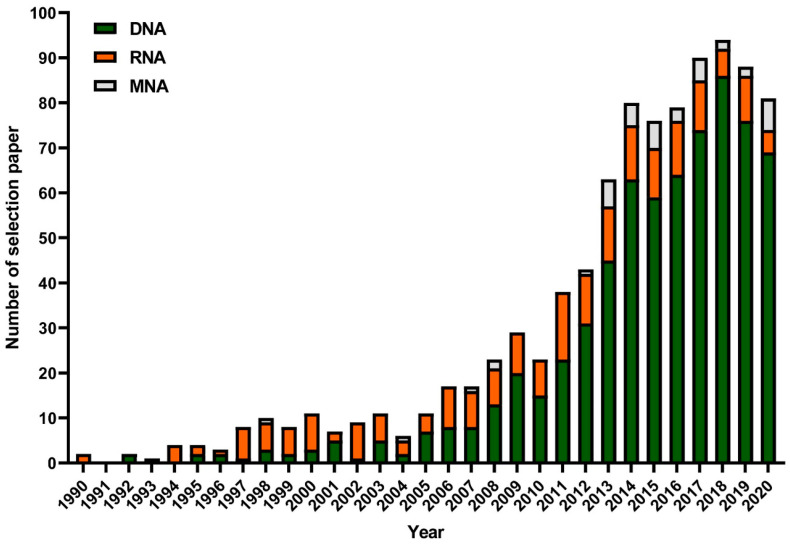
The annual distribution of aptamer selection studies (DNA, RNA, and MNA) [17].

The application of aptamers in biosensing is limited by the variety of aptamers targeting small molecules. Qian et al. [16] provided an analysis of the annual trends in aptamer selection studies involving RNA, DNA, and modified nucleic acids (MNAs) from 1990 to 2020. Between 1990 and 2007, the SELEX method primarily focused on RNA analogs, as depicted in Figure 2, which likely reflects the early belief that RNA had functional advantages over DNA [17]. However, since 2008, there has been a significant increase in studies using DNA analogs in SELEX, with the number of such studies peaking in 2018. In Phase III, DNA aptamer SELEX dominates the field, while the publication rate for RNA aptamer SELEX remains relatively constant.

Both DNA and RNA aptamers are capable of binding to their targets with high specificity and selectivity. In the early stages of SELEX, RNA libraries were predominantly used because RNA has a greater propensity to form complex tertiary structures [18]. The additional hydroxyl group at the 2′ position of ribose allows RNA to adopt more stable three-dimensional shapes, fostering strong RNA–RNA interactions. However, this same hydroxyl group can also make RNA more prone to hydrolysis through base-catalyzed nucleophilic attacks on the adjacent phosphorus atoms in the phosphodiester backbone. For example, RNA aptamers targeting keratinocyte growth factor degrade rapidly in human serum, with up to 90% degradation occurring within seconds [19]. As a result, researchers shifted toward using single-stranded DNA in SELEX experiments, as DNA is more chemically and biologically stable than RNA. Additionally, DNA-based SELEX is more cost-effective and efficient since RNA SELEX requires time-consuming steps like in vitro transcription and reverse transcription for each round of selection. The process of selecting DNA aptamers is also less labor-intensive compared to RNA aptamers. While standard DNA and RNA remain widely used for aptamer selection [8], chemical modifications to natural nucleic acids can significantly enhance their functionality. These modifications can produce aptamers with improved stability and performance in biological environments.

### 2.3. Superiority of Aptamers

Aptamers are often compared to antibodies, as both have properties as affinities [20]. However, aptamers have unique advantages over antibodies and other protein-based affinity reagents [21,22,23,24]: (1) Aptamers can be produced at a larger scale than antibodies, and the retained genetically encoded aptamer sequences can be expressed in vivo or in cultured cells. (2) Because aptamer production is based on a chemical process, rather than a biological process, it avoids the problems of viral or bacterial contamination that can occur during antibody production and reduces the potential for batch-to-batch variability, thus improving the stability and consistency of the product. (3) As therapeutic agents, aptamers typically exhibit fewer immune responses than proteins, and their smaller size allows them to enter biological regions that are difficult for antibodies to reach (typically aptamers of less than 30 kDa and antibodies around 150 kDa). (4) In addition, aptamers are easily chemically modified, which helps optimize their pharmacokinetic properties, such as reducing their renal clearance and extending their half-lives. (5) Extending the storage time at room temperature may cause the aptamer to unfold. However, this characteristic does not affect its functionality because the aptamer can be refolded into its functional state through a simple heating and cooling annealing procedure in an appropriate buffer, thereby addressing the cold chain issue and reducing transportation costs. (6) Since aptamers are oligonucleotides, they can serve as tools for other techniques involving nucleic acid systems (such as amplification technologies, DNA nanotechnology, or DNA computing), thus expanding their potential in biological research and applications.

Small molecules have a smaller surface area, fewer chemical groups, and simpler structures compared to larger targets like proteins. As a result, the interactions between small molecules and aptamers are generally weaker and fewer in number, leading to a lower binding affinity and less specificity in the aptamer-target interaction. Moreover, the process of isolating aptamers often requires attaching small molecules to solid supports, such as beads, to help separate the aptamers that bind to the target from those that do not. This process is straightforward for proteins, as they can easily be conjugated to surfaces using standard chemical methods, allowing most of the protein’s surface to be available for binding [25]. In contrast, conjugating small molecules to surfaces is more challenging and involves additional complexities. Small molecules are chemically more diverse than proteins because they can contain a wider variety of functional groups. As a result, specialized conjugation methods or complex chemical synthesis are often needed to attach them to a surface or other materials. However, small molecules usually have only a limited number of functional groups, which restricts their ability to form stable conjugates and reduces their interaction with aptamers immobilized on surfaces [26]. In fact, using linkers or other conjugation elements to attach small molecules can significantly change their chemical and biological properties, leading to a dramatic reduction or even complete loss of affinity between aptamers selected against the conjugated target and the free target [27]. Moreover, the low molecular weight and the lack of charged groups in small molecules make it more difficult to directly separate target-aptamer complexes from free aptamers using certain techniques, such as capillary electrophoresis-SELEX (CE-SELEX), which is highly efficient when applied to protein targets [28].

### 2.4. Advances in Screening of Small-Molecule Aptamers

**Figure 3:**
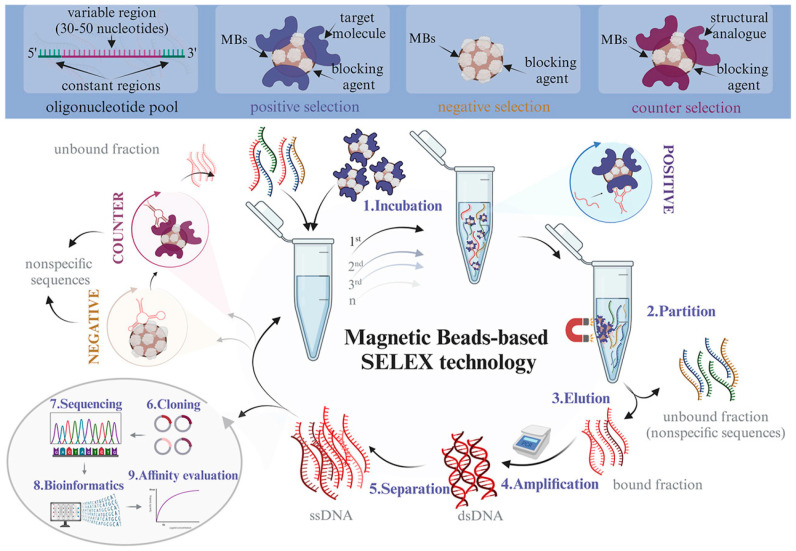
Schematic representation of classic MBs-SELEX technology [28].

Magnetic bead-based nonhomogeneous SELEX technology (MBs-SELEX) has been widely employed to isolate aptamers for hundreds of small-molecule targets, including drugs [29], steroids [30], nucleotides [24], and antibiotics [31,32]. This process typically involves 10–30 rounds of screening with nanomolar to micromolar affinities. The use of paramagnetic beads as a solid support for the immobilization of target molecules presents a number of advantages. These include the ability to partition samples in a rapid and straightforward manner through the application of a magnetic field, the ability to perform rigorous cleaning with minimal risk of loss, and the possibility of PCR amplification directly on the surface of the beads. Furthermore, the use of paramagnetic beads allows the immobilization of target molecules in small sample volumes, typically within the microlitre range (50–250 μL) [27]. The conventional MBs-SELEX methodology is depicted in schematic form in Figure 3 [28]. However, background adsorption of the library strand to the magnetic bead surface or linker can also result in the enrichment of nonspecific binders, which may prolong the SELEX process and potentially lead to screening failure.

**Figure 4:**
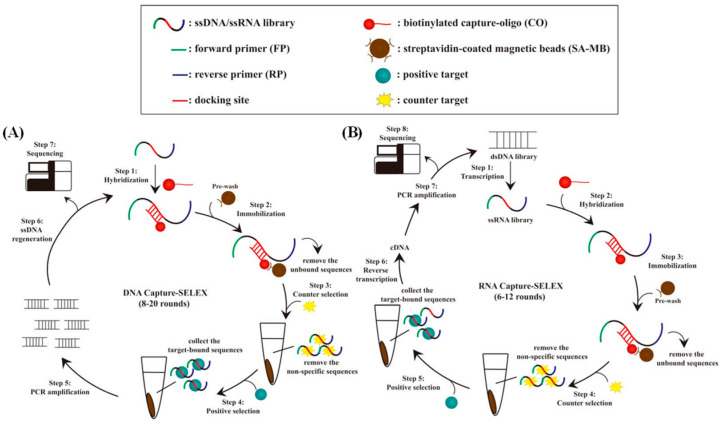
(**A**) The procedure of DNA Capture-SELEX [34]. (**B**) The procedure of RNA Capture-SELEX [34].

In light of these constraints, a modified SELEX approach, designated ‘capture-SELEX’, has gained considerable traction. This technique employs short, bead-bound complementary DNA (cDNA) sequences hybridized to specific regions of the library strand [33], which are immobilized on the surface of the beads to generate DNA/RNA analogs capable of binding to small molecules [34]. The classical procedure of the DNA Capture-SELEX selection method comprises the following steps: hybridization, fixation, elution of target-binding sequences, polymerase chain reaction (PCR) amplification, single-stranded DNA (ssDNA) regeneration, and sequencing. These steps are illustrated in Figure 4A. RNA Capture-SELEX employs the same selection strategy as that of DNA Capture-SELEX, with the exception of two additional transcription and reverse transcription steps. These are shown in Figure 4B. The two nonhomogeneous SELEX methods described here impose spatial constraints on the interaction between the bead-bound target or library molecule and its binding partner, thereby limiting the efficacy of the process. These factors collectively reduce the probability of successful aptamer isolation and limit the binding affinity of the aptamer to the target.

**Figure 5:**
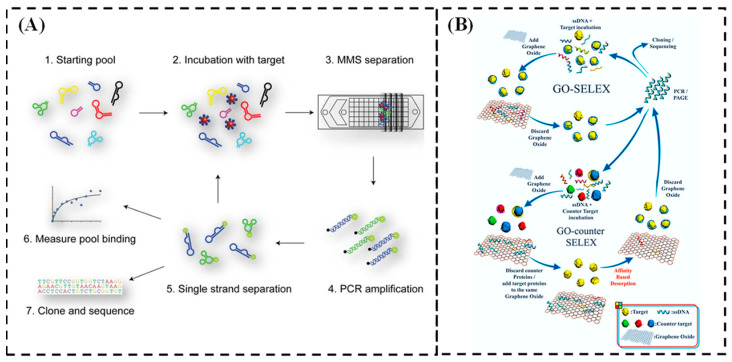
(**A**) Operational diagram of the microfluidic SELEX prototype device [37]. (**B**) Schematic illustration of the steps involved in the GO-SELEX procedure of aptamer identification [36].

Microfluidic SELEX (M-SELEX) represents a combination of traditional SELEX with a chip-based microfluidic system, as illustrated in Figure 5A [35]. Compared to conventional bead-based techniques, microfluidic SELEX employing beads necessitates a reduced quantity of material, is less labor intensive, and mitigates the likelihood of nonspecific binding. Despite the evident advantages, M-SELEX platforms are not widely employed due to the necessity for specialized electronics and microfluidic apparatus [36]. Nevertheless, microfluidic SELEX (M-SELEX) enables the efficient selection of high-affinity aptamers through the miniaturization of the SELEX process.

Graphene oxide (GO) is a heterogeneous compound composed of oxygen-functionalized derivatives, specifically a single layer of carbon atoms with oxygen-containing functional molecules. Under aqueous conditions, graphene oxide (GO) demonstrates good biocompatibility, allowing single-stranded DNA (ssDNA) or RNA to adsorb onto the GO surface through interactions with water and π-π stacking. The graphene oxide-based SELEEX screening method (GO-SELEX) is used to select aptamers that bind with high affinity and specificity to the Nampt protein, as shown in Figure 5B [38]. This method reduces the number of complex steps involved in traditional SELEEX, enhances the efficiency of the screening process, and helps identify aptamers with high affinity and specificity in a shorter period of time.

**Figure 6:**
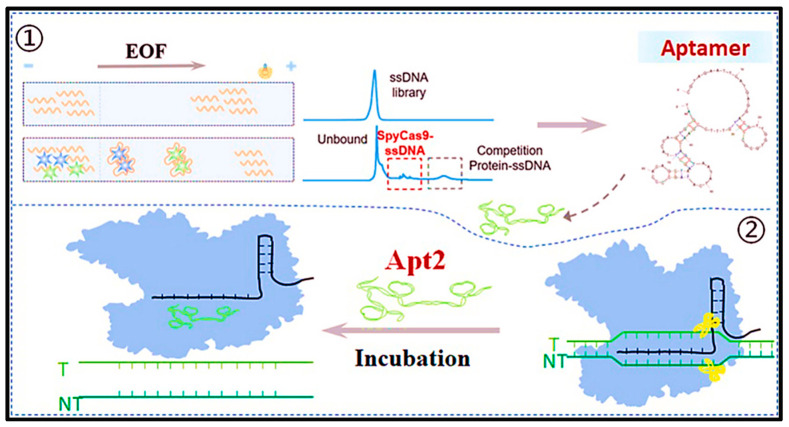
Schematic representation of the principle of CE-SELEX [38].

The initial studies on aptamers employed methodologies based on nitrocellulose filtration, exemplified by the seminal SELEX experiments conducted by Tuerk and Gold. In this technique, the targets and nucleic acid libraries were initially incubated in solution before being introduced into a nitrocellulose filter. As neither the target nor the aptamer is immobilized on a surface and both are able to move freely in solution, this method is considered to be homogeneous. The most prominent homogeneous SELEX platform is CE-SELEX, which employs the differential mobility of unbound and target-bound library molecules in the presence of an external electric field to separate them based on their charge and size [39]. This technique has been successfully employed for the separation of aptamers targeting a number of protein molecules, as illustrated in Figure 6. The aptamer Apt2, which has a high affinity for the SpyCas9 protein, was successfully screened by capillary electrophoresis (CE) combined with the exponential enrichment technique of systematic evolutionary ligand (SELEX) in a round of pressure-controlled selection (OPCS). This has been shown to be an extremely efficient method of inhibiting the activity of SpyCas9, with a rapid onset of action and low dose. Therefore, it provided a promising solution for the CRISPR-Cas9 system, offering a new and potential strategy for precise control of the CRISPR-Cas9 system.

### 2.5. Challenges for Small-Molecule Aptamers

**Figure 7:**
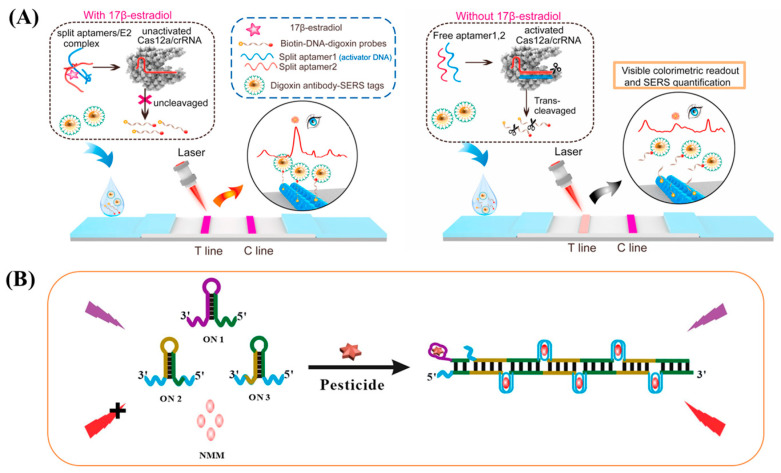
(**A**) Principle of split aptamers regulated CRISPR-SERS LFA for 17β-estradiol detection. T line and C line represent the test line and control line of the strip, respectively [40]. (**B**) Graphic expression for label-free, enzyme-free, and signal-on analysis of profenofos based on target-switched HCR and the specific intercalation of NMM in G-quadruplex DNA [40].

Despite the existence of aptamer technology for more than two decades, the development of novel aptamers capable of binding to practical small-molecule targets has remained a significant challenge due to the inherent difficulties associated with small-molecule aptamer creation [8]. The limitations of aptamer development can be attributed to the high cost of reagents, the labor-intensive nature of the process, the slow speed of manipulation, and the low success rate of aptamer creation, particularly for those with high binding affinity and specificity, which are significant challenges [9]. Therefore, it is evident that the future success of aptamers in a range of fields, including biomedicine and food safety, will depend on the ability to overcome these significant challenges. In recent years, as illustrated in Figure 7A, Li et al. [40] have developed a detachable aptamer that uses 17 β-estradiol to specifically bind complementary CRISPR/Cas12a RNAs, thus competitively modulating the reverse cleavage ability of CRISPR/Cas12a. This design is based on the rapid development of CRISPR-assisted nucleic acid-targeted LFA strategies and is known for its remarkable sensitivity and specificity. As illustrated in Figure 7B, Chen et al. [41] designed a pioneering aptamer-based fluorescent system for target-initiated hybrid chain reaction (HCR)-assisted signal amplification and specific embedding of N-methylmesoporphyrin IX (NMM) in G-quadruplex DNA. This system uses target-initiated HCRs for the label-free, enzyme-free, and highly sensitive detection of pesticides (profenofos). The advancement of biotechnology and computational technology will facilitate the refinement of screening methods for small-molecule aptamers, enhancing their precision and efficiency. This will enable them to realize greater potential in practical applications. Adaptamers, as highly specific recognition elements, will facilitate the advancement of biomedicine, environmental monitoring, and industrial applications, thereby contributing to human health and social progress.

## 3. Progress of Surface-Enhanced Raman Spectroscopy

### 3.1. Advancements in Surface-Enhanced Raman Spectroscopy

Raman scattering (RS) is a form of inelastic scattering: when incident light illuminates an object, most scattered light retains the same frequency as the incident light. However, there is a very small amount of scattered light that causes energy exchange between photons and material molecules as a result of inelastic collisions, which causes the frequency of photons to change. This small portion of scattered light is known as Raman scattering [42].

**Figure 8:**
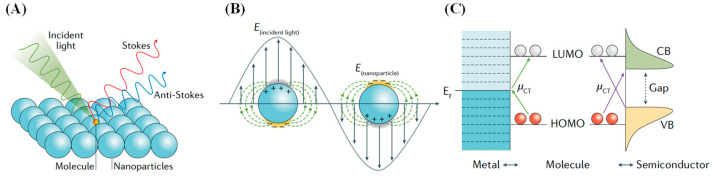
Schematic representation of SERS and its enhancement mechanisms [45]. (**A**) SERS. (**B**) Electromagnetic enhancement mechanism. (**C**) Chemical enhancement mechanism.

Because of the low probability that Raman scattering occurs, traditional Raman spectroscopy signals are weak and easily affected by fluorescence signals from target molecules. As a result, early applications of Raman spectroscopy were limited, and the method was not widely adopted. However, in 1974, British physicist Fleischmann et al. [43] discovered the phenomenon of Surface-enhanced Raman scattering (SERS) (Figure 8A), in which rough metal surfaces significantly enhance the Raman signals of molecules adsorbed on their surfaces, with amplification factors reaching 10^4^ to 10^6^ times [44]. This discovery led to the development of SERS, which not only preserves the rich “fingerprint” information of traditional Raman spectroscopy but also offers higher signal intensity and excellent sensitivity, greatly promoting the development and application of Raman spectroscopy.

To date, there is still no consensus on the SERS enhancement mechanism, with two primary theories: physical enhancement and chemical enhancement, as shown in Figure 8B,C, with physical enhancement considered the dominant factor [45]. Physical enhancement refers to electromagnetic enhancement, where the localized field strength increases due to plasma resonance excitation, also known as the “hot spot” effect, resulting in significant signal enhancement for molecules adsorbed on rough metal surfaces. On the other hand, the chemical enhancement mechanism attributes the enhancement to charge transfer and increased polarizability, leading to resonance-like amplification.

SERS, as a high-sensitivity and rapid spectroscopic detection technique, has been widely applied in the qualitative and quantitative analysis of small molecule residues. By combining the high sensitivity of SERS technology with the specific recognition ability of aptamers, numerous aptamer-based SERS sensors have been developed for detecting various small molecules. For example, Liu et al. utilized electrostatic interactions to adsorb Cy3-labeled tetrodotoxin aptamers onto the surface of AuNPs@MIL-101 substrates. Cy3 exhibited a strong SERS signal, and when the target toxin was introduced, the specific binding of the aptamer to the toxin caused a conformational change, leading to detachment from the substrate surface and a decrease in the SERS signal intensity of Cy3 [46], achieving an ultra-high detection sensitivity of 8 pg/mL. Wei et al. conjugated an atrazine aptamer and the Raman reporter molecule 4-MMBN onto silver nanoparticles (MMBN-AgNPs-Apt) as the Raman probe, and linked the complementary strand of the aptamer to Fe_3_O_4_/Au core–shell nanoparticles (Fe_3_O_4_@Au-cDNA) as the Raman substrate. The specific binding of atrazine to its aptamer hindered the formation of Fe_3_O_4_@Au-AgNPs complexes, resulting in a decrease in the SERS signal intensity of MMBN as the atrazine concentration increased, with a detection limit as low as 0.67 nM [47]. In 2022, Lu et al. [48] used gold nanoparticles as a SERS substrate for the simultaneous detection of influenza A (H1N1) and the novel coronavirus, with clinical trials showing that the false negative rate was significantly lower than that of colloidal gold immunochromatography.

### 3.2. Advances in SERS Substrates

The SERS substrate is critical for achieving signal enhancement in SERS, as the performance of Surface-enhanced Raman spectroscopy is largely dependent on the material selection and structural design of the substrate. In recent years, researchers around the world have focused on developing efficient and stable SERS substrate materials. By continuously optimizing substrate structures and synthesis methods, the enhancement capability of SERS substrates has been improved. Common SERS substrates include metal nanoparticles and their composites, semiconductor materials, and flexible substrates.

**Figure 9:**
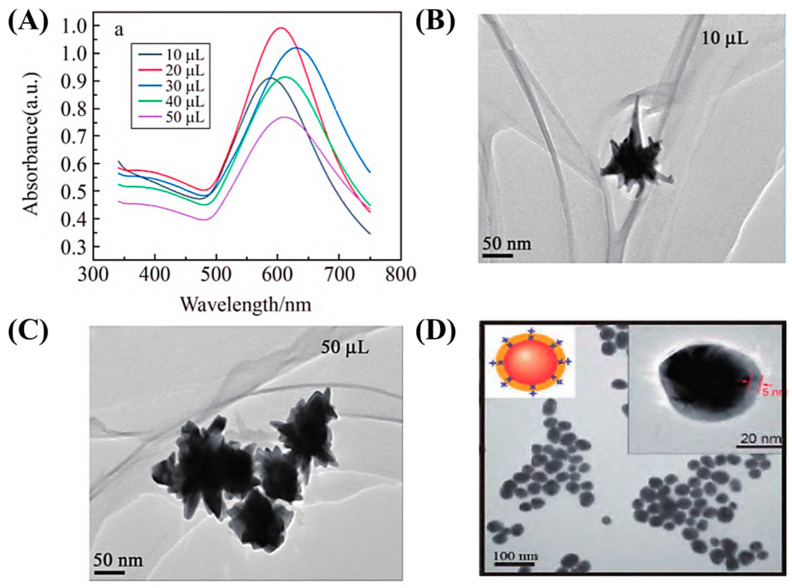
Ultraviolet absorption spectrum of gold nanostars (Au NS) [50] (**A**) and TEM images (**B**,**C**). (**D**) Silver-shell gold-core nanoparticles (Au@Ag NPs) [51].

Gold nanoparticles, known for their chemical stability and biocompatibility, are widely used in SERS substrates. Haynes et al. [49] developed Ag NP lattices using nanoimprint technology, controlling nanoparticle size and shape. Maneeprakorn et al. [50] synthesized gold nanostars (Au NS) using a hydroquinone reduction method, featuring numerous short, sharp branches that significantly enhanced the “hot spot” effect at the tips (Figure 9A–C). However, due to the high cost of gold, recent research has shifted toward exploring silver nanoparticles and their composite structures to reduce costs. In 2018, Jia et al. [51] synthesized gold-core silver-shell nanoparticles (Au@Ag NP) using a thermal reduction method (Figure 9D). This core–shell structure combines the stability of gold with the high SERS activity of silver and can balance the enhancement and stability of the signal by adjusting the core–shell ratio.

Semiconductors, with their excellent optoelectronic properties and diverse structures, can adapt to SERS effects. Ni et al. [52] prepared uniform ZnO nanoparticles using thermal decomposition of zinc acetate, modified with molecules, such as thiophenol (TP), p-nitrothiophenol (PNTP) and p-aminothiophenol (PATP), and conducted Raman detection under different laser wavelengths. The study found that the SERS enhancement increased with photon energy. Flexible SERS substrates are constructed by fixing nanoparticles on flexible materials to create multiple hot spots that enhance Raman signals. These substrates, with their excellent flexibility, can better conform to samples and are particularly advantageous when dealing with irregular sample surfaces. Tang et al. [53] developed flexible SERS substrates using inkjet printing, studying the effects of varying silver ink concentrations and print layers on detection sensitivity and testing the substrate’s performance on curved surfaces. In addition, Qiu et al. [54] utilized graphene-metal nanoparticle composite structures as SERS substrates. Graphene not only provided additional enhancement but also prevented copper nanoparticles from oxidizing. These innovative materials have laid the foundation for the widespread application of SERS technology and have facilitated the development of more efficient multiplex detection techniques.

### 3.3. Progress of Labeled Raman Substrates

**Figure 10:**
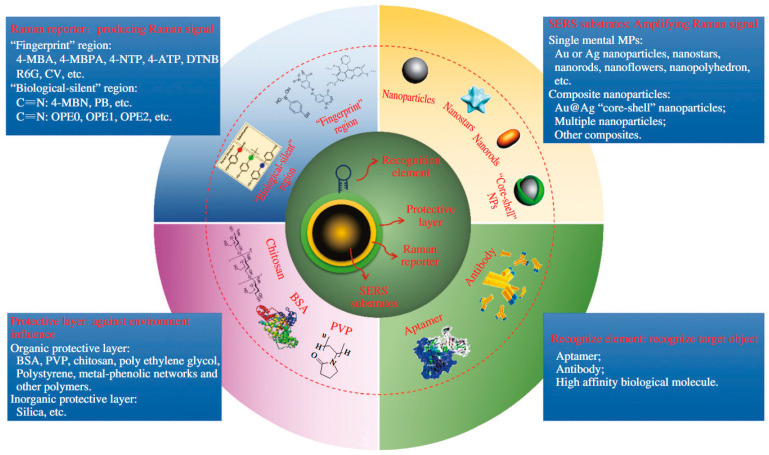
Overview of the general composition, structure, and design of SERS-tags, together with the function of each component [55].

Labeled Raman substrates involve the adsorption of specific labeling molecules onto the Raman substrate, enabling selective detection and analysis of target molecules. This technology is widely applied in fields such as biological detection, chemical analysis, and environmental monitoring. The choice of labeling molecules directly impacts the sensitivity and specificity of the Raman substrate. Different labeling molecules can satisfy different detection requirements, significantly improving the sensitivity to target molecules, enhancing signal selectivity, and reducing background noise. The chemical structure and spectral properties of the labeling molecules determine the intensity and resolution of the Raman signals. Common labeling molecules include dyes and biomolecules (Figure 10).

Dye labeling involves the use of Raman-active dyes (e.g., Rhodamine 6G) to recognize target molecules, producing Raman signals on the SERS substrate. These dye-labeled molecules typically adsorb onto metal nanoparticles and rely on chemical enhancement mechanisms to significantly enhance signals, effectively improving SERS detection sensitivity. Cao et al. [56] designed nanoparticle probes labeled with Raman dyes, which exhibit affinity for specific proteins. These probes, combined with Surface-enhanced Raman scattering spectroscopy (SERS), were used to screen protein-small-molecule interactions and protein–protein interactions in a multiplex protein array format. Anne März et al. [57] proposed a new method for detecting labeled Raman substrates. Using fluorescent dye FR-530 as an antibiotic labeling molecule, the target antibiotic binds directly to the labeled molecule in the presence of the target, allowing detection and quantification of erythromycin by analyzing the SERS spectrum of the fluorescent dye.

Biomolecule labeling employs specific molecular probes, such as antibodies or aptamers, to bind to the target for detection. This method relies on the specificity of molecular probe-target interactions [58], with SERS substrates further amplifying the signal. Dinish et al. [59] designed ultra-sensitive nanoprobe tags using SERS for multiplex detection of biomarkers, demonstrating their potential to monitor tumor progression and treatment, creating novel theranostic probes. Wang et al. [60] proposed a biosensor based on self-assembled AuNR arrays for the specific detection of exosomes secreted by SK-Br-3 cells.

**Figure 11:**
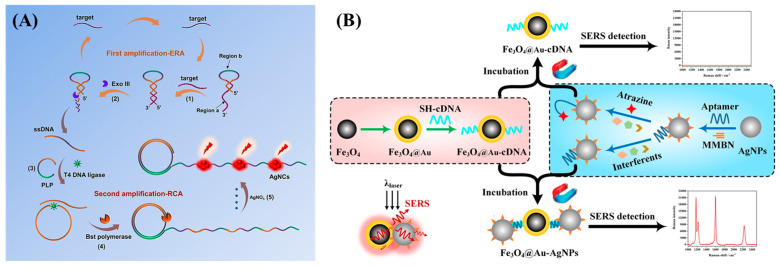
(**A**) Scheme for the homogeneous DNA machine based on Exo III-assisted RCA cascade amplification for the intelligent detection of HIV/HCV DNA [61]. (**B**) Schematic representation of the principle for the analysis of atrazine based on the SERS aptasensor [47].

Labeled Raman substrates, with their high sensitivity and specificity, have shown the capability to precisely detect target molecules in complex samples. As a result, they are widely applied in biomolecular markers, medical diagnostics, and environmental pollutant monitoring. One of the most common methods for virus detection is PCR amplification. However, PCR requires complex nucleic acid amplification processes and is time consuming, which is not ideal for rapid large-scale detection. To address this, Wu et al. [61] proposed a novel SERS sensor based on silver nanorods (AgNR) ultra-sensitive detection of SARS-CoV-2 RNA (Figure 11A). Using a portable Raman spectrometer, SERS measurements were made, allowing rapid and accurate detection of SARS-CoV-2 infection in the early stages. Common pesticide residue detection methods include mass spectrometry and chromatography, which are known for their precision and accuracy. However, these methods require specialized operation and expensive equipment, making them unsuitable for on-site detection. Wei et al. [47] developed an MMBN-AuNPs-aptamer probe with AgNPs as the SERS enhancement substrate, achieving the detection of atrazine in cherry tomatoes and grapes with a high recovery rate (Figure 11B).

### 3.4. Advances in Raman Signal Processing with Deep Neural Networks

**Figure 12:**
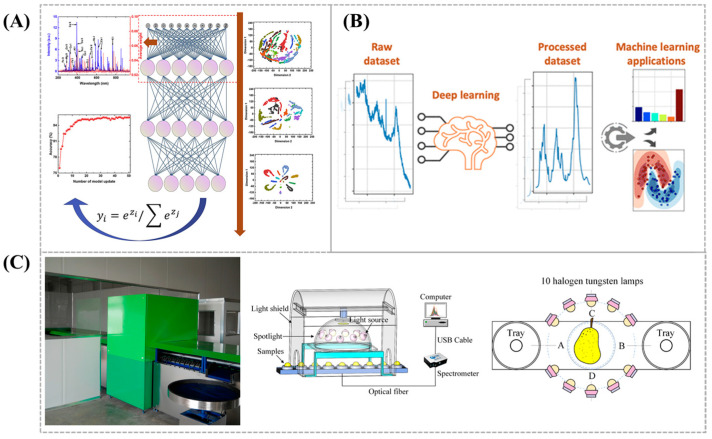
(**A**) Schematic diagram of LIBS combined with the DNN model [62]. (**B**) Schematic diagram of cascaded deep convolutional neural networks (CNNs) [63]. (**C**) Schematic diagram of the dynamic online acquisition device of Yali pears [64].

Because of the complexity of sample components, Raman spectra often exhibit multiple peaks, making accurate differentiation and analysis challenging. Therefore, signal processing algorithms must meet high standards of accuracy and real-time performance. In recent years, deep learning technology has rapidly developed, with deep neural networks (DNNs) demonstrating excellent feature learning capabilities, enabling the discrimination of subtle differences between features and thus accurately identifying subtle differences in spectral data. Currently, deep neural network-based spectral processing methods have been widely applied in soil analysis [62] and food detection [63] (Figure 12A,B).

In raw Raman spectra, various types of noise interference often exist, such as the sample’s own fluorescence signals, offsets, and baseline curvature. Traditional methods for Raman spectral denoising, such as filtering and baseline correction, may result in the loss of critical spectral information. In contrast, deep neural networks can effectively extract useful signals from noise, significantly improving the signal-to-noise ratio of Raman spectra while preserving spectral integrity. For example, Kazemzadeh et al. [64] developed a deep learning method capable of preprocessing raw Raman spectra without any manual input (Figure 12C). First, a deep convolutional neural network (CNN) was trained on randomly generated spectra with defects, and then tested using simulated Raman spectra, SERS imaging, and Raman spectra of human bladder cancer tissues. Finally, the method classified SERS spectra of human placenta extracellular vesicles. This approach enables one-step spectral preprocessing, offering faster processing speed and higher accuracy.

Deep neural networks can extract features from large volumes of Raman spectral data to identify and classify chemical components in complex mixtures. This meets the need for qualitative analysis of complex spectral data and provides higher accuracy and robustness in the classification and identification of multiple components. Liu et al. [65] proposed a solution for chemical substance identification, training a CNN to automatically recognize substances in Raman spectra without preprocessing. At the same time, Fan et al. [66] introduced a new method called Deep CID (Deep Learning-based Component Identification) for qualitative analysis of Raman spectra. Using deep learning, this method identifies components in mixtures and constructs CNN models for each compound in the database. It extracts useful information from the raw Raman spectra and identifies the components within the mixture.

Deep learning can model the complex relationship between spectra and concentration. By constructing nonlinear models, deep neural networks can accurately predict the concentration of chemical substances from Raman spectra, enabling quantitative analysis. Compared to traditional methods, deep neural networks offer higher precision in quantitative analysis, particularly when dealing with complex components or overlapping signals. For example, Pian et al. [67] proposed a single-layer shallow convolutional neural network structure combined with an elastic net for quantifying glucose concentration in blood using Raman spectra. Similarly, Weng et al. [68] developed a deep learning network for quantitative identification of SERS, achieving 98.05% drug recognition accuracy in urine, which provided higher analytical efficiency and accuracy compared to traditional machine learning methods.

## 4. Application of Aptamer-Based SERS Technology

### 4.1. Application of Aptamer-Based SERS Technology in Pesticide Detection

In recent years, with growing public concern about the safety of agricultural products, there is an urgent need to develop rapid detection technologies, such as rapid screening on-site of high-quality fruits and vegetables, instant detection of agricultural product freshness and composition, real-time monitoring of plant growth and diseases, and quick detection of harmful chemical residues, including pesticides. Surface-enhanced Raman scattering (SERS) is an emerging ultrasensitive detection method known for its simplicity, speed, and high sensitivity. In particular, with advances in nanotechnology, SERS has garnered special attention and is widely used in the field of food safety [69,70,71,72]. Aptamers, developed through the exponential enrichment method (SELEX), are short single-stranded oligonucleotides (RNA or DNA) that can selectively bind to targets with high affinity [73,74,75]. Compared to antibodies, aptamers have advantages such as high specificity, low molecular weight, broad target range, and ease of synthesis and modification, making them ideal alternatives for developing biosensors to detect pesticide residues [76,77,78].

**Figure 13:**
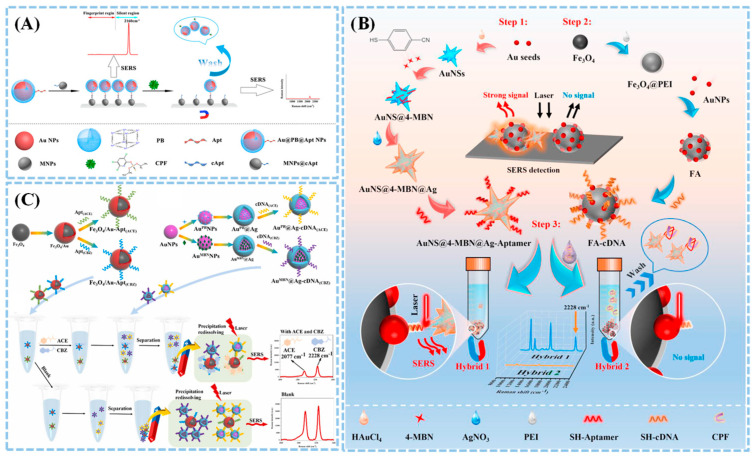
(**A**) Schematic diagrams of SERS aptasensor for different pesticide detections [79]. (**B**) SERS aptasensor technology for CPF detection based on bimetallic nanolabels and magnetic substrates [80]. (**C**) SERS aptasensor technology for simultaneous detection of ACE and CBZ mixed pesticides using Raman silent spectral window labeling molecules [81].

Monitoring pesticide residues on the surfaces of fruits and vegetables in a nondestructive and noninterfering manner has remained a research focus in recent years. For example, Wang et al. [79] proposed an interference-free aptamer sensor based on SERS for the trace detection of chlorpyrifos (CPF) in cucumbers and pears, as shown in Figure 13A. This aptamer sensor utilizes gold nanoparticles coated with Prussian blue (Au@PB NPs) as SERS tags, providing a unique and strong Raman peak at 2160 cm^−1^, which not only avoids overlapping with the Raman spectra of real samples in the 600–1800 cm^−1^ range but also enhances the sensor’s resistance to matrix effects. Under optimal conditions, this sensor demonstrates a linear response to CPF detection in the range of 0.1–316 ng/mL, with a detection limit of 0.066 ng/mL. The aptamer sensor shows excellent applicability in determining CPF in cucumber, pear, and river water samples, featuring non-interference, specificity, and sensitivity, with recovery rates closely correlated with high-performance liquid chromatography–tandem mass spectrometry (HPLC-MS/MS), providing an effective detection strategy for other pesticide residues. Additionally, Ma et al. [80] introduced a high-performance SERS aptamer sensor combining nanotags (AuNS@4-MBN@Ag-aptamer) with magnetic substrate cDNA-Fe_3_O_4_@Au NPs (Figure 13B). This sensor uses star-shaped bimetallic nanotags as the main Raman signal enhancement material, with 4-mercaptobenzonitrile (4-MBN) serving as a “biosilencing zone” reporter gene (2228 cm^−1^). Under optimal SERS conditions, the aptamer sensor exhibits a wide linear detection range from 2.5 × 10^2^ to 5.0 × 10^4^ pg/mL, with a low detection limit of 220.35 pg/mL for wheat and apples. Thus, aptamer sensors based on high-performance “biosilencing zone” nanotags combined with magnetic substrates are promising tools for monitoring trace CPF in complex matrices.

Ma et al. [81] achieved the specific simultaneous detection of acetamiprid (ACE) and carbendazim (CBZ) in complex sample matrices using two Raman labeling molecules (PB and MBN), as illustrated in Figure 13C. They synthesized two Raman nanoprobes by adding MBN and PB into the Au-Ag core–shell gaps and coupling them with two complementary DNA (cDNA) strands. Furthermore, modified Fe_3_O_4_/Au nanoparticles with two aptamers were used as capture molecules. The results indicated that Raman intensities at 2077 cm^−1^ and 2228 cm^−1^ exhibited opposite trends with respect to ACE and CBZ concentrations, with detection limits as low as 9.43 μg/kg for ACE and 9.17 μg/kg for CBZ. Wei et al. [82] employed flexible film materials as SERS substrates, using AuNS@Ag to create a uniform and highly active SERS substrate through liquid–liquid self-assembly on PVDF/CQD films. During the detection process, the aptamers specifically captured CBZ molecules, while the nitrile-mediated Raman label (MMBN) linked to AuNP provided optical anti-interference signals. This technique allowed the effective detection of carbendazim (CBZ) in apple peels, achieving concentrations as low as 1.20 ng/cm^2^, which is significantly below the maximum residue limit for CBZ in apples.

### 4.2. Application of Aptamer-Based SERS Technology in Veterinary Drug Residue Detection

In recent years, veterinary drug residues in food safety have become a key focus of social concern. Although regulations on the allowable levels of veterinary drug residues in food have been established in China, some practitioners continue to misuse prohibited drugs, disregarding withdrawal periods, overusing drugs, or using banned substances illegally. This leads to the accumulation of drug residues in animal tissues or organs, which can enter the human food chain and pose health risks [83,84]. Surface-enhanced Raman spectroscopy (SERS) offers advantages such as high sensitivity, accuracy, and low equipment dependence [85]. Compared to methods such as enzyme-linked immunosorbent assays, colloidal gold immunochromatography, and chromatography, SERS shows significant application advantages in the detection of residues of veterinary drugs in food.

**Figure 14:**
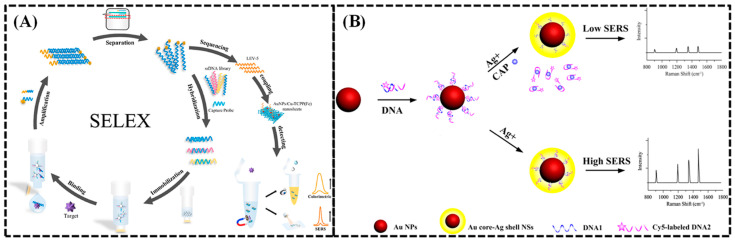
(**A**) Schematic diagram for the detection of levamisole using SERS-SELEX technology [89]. (**B**) Schematic diagram of SERS-active Au@Ag nanostars for CAP detection [87].

For example, levamisole (LEV) is a veterinary drug that commonly residues in animal-based food products. Consuming products with high levels of LEV can cause a range of harmful health effects. Li et al. [86] utilized Capture-SELEX technology to select high-affinity and high-specificity aptamers for LEV (Figure 14A). By combining SGI dye detection and ITC affinity and specificity results, they identified the LEV-5 sequence with favorable affinity and specificity. Additionally, they synthesized two-dimensional AuNPs/Cu-TCPP(Fe) nanostructures with the dual characteristics of peroxidase-like enzyme activity and Raman signal. Based on the selected aptamer and the prepared dual functional Cu-TCPP(Fe) nanoparticles, they developed a colorimetric Surface-enhanced Raman spectroscopy (colorimetric-SERS) dual-mode aptamer sensor for detecting LEV, achieving a detection limit as low as 1.12 nM. This aptamer-based method was also successfully applied to detect LEV in milk, with recovery rates ranging from 94.95% to 111.2%, providing potential applications for detecting harmful substances in food. Yan et al. [87] utilized gold nanoparticles as a SERS-enhanced substrate in their study on chloramphenicol detection (Figure 14B). They developed a fluorescent dye Cy5-aptamer-gold nanoparticle recognition probe, where the Cy5-aptamer binds to chloramphenicol to form a stem-loop structure that separates from the gold nanoparticles, leading to a significant reduction in SERS signal intensity. This achieved high sensitivity detection of chloramphenicol residues (CAPs) in milk samples, with a detection limit of 0.19 ng·L^−1^ and a recovery rate of 96.6% to 110.2%. Fei et al. [88] established a SERS-aptamer sensor to simultaneously detect chlorpromazine and melamine in milk. After the aptamer binds with chlorpromazine and melamine, they controlled the reduction of silver nanoparticles on the surface of the aptamer, forming a Cyr/Mel-DNA-Ag NPs composite to enhance the Raman effect. Under optimal detection conditions, the linear detection range for chlorpromazine was 0.10 to 0.65 mg·L^−1^, with a detection limit of 13.6 μg·L^−1^; for melamine, the linear range was 0 to 0.5 mg·L^−1^, with a detection limit of 43.5 μg·L^−1^. This provides a reference for the rapid detection of veterinary drug residues in milk. In summary, the application of SERS combined with aptamer detection in animal food safety is widespread and effective, showing promising prospects for future development in the detection of veterinary drug residues.

### 4.3. Application of Aptamer-Based SERS Technology in Antibiotic Detection

Antibiotics are a class of natural or semi-synthetic chemical substances with broad-spectrum antibacterial properties. They include various types, such as β-lactams, tetracyclines, aminoglycosides, fluoroquinolones, and macrolides [90], and are widely used in the prevention and treatment of various diseases [91,92]. However, the abuse of antibiotics can lead to environmental pollution and pose a serious threat to human health and ecological safety. Furthermore, it may result in the emergence of ’superbugs’, which limits the clinical application of antibiotics [90]. The high consumption of antibiotics and improper disposal or excessive discharge can easily lead to their accumulation in environmental media such as water and soil. Therefore, the rapid, sensitive, and real-time detection of antibiotics in food and the environment is of great significance for the protection of the ecosystem and human health. Traditional detection methods are time-consuming and require specialized instruments, which often cannot meet the detection demands. In recent years, biosensors have been widely used as an effective alternative for antibiotic detection. Among them, SERS-biosensor using aptamers as molecular recognition elements have attracted significant attention due to their high stability and strong specificity.

**Figure 15:**
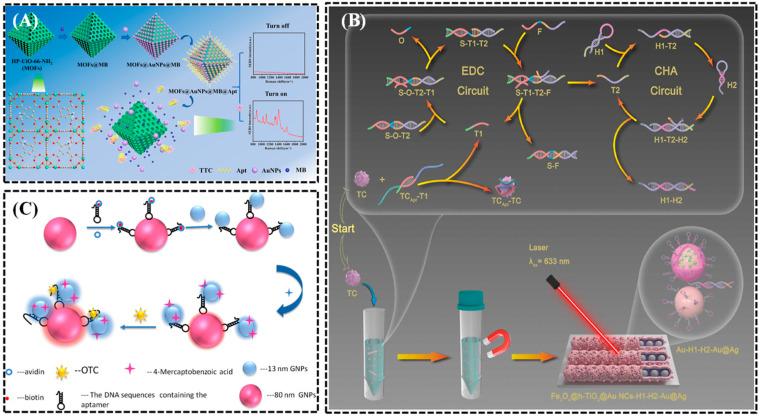
(**A**) Schematic diagram of a SERS sensor for tetracycline detection based on aptamer-gated HP-UiO-66-NH_2_ with target-responsive release [94]. (**B**) Schematic diagram of a SERS aptasensor for tetracycline detection based on aptamer recognition and cascade DNA network amplification [95]. (**C**) Schematic diagram of a SERS-based nanobiosensor for the detection of tetracycline [96].

For example, tetracycline (TTC) is a broad-spectrum antibiotic that is widely used in medicine, livestock, and aquaculture due to its low cost, spectral properties, and minimal side effects [93]. However, in recent decades, the overuse of tetracycline has led to an increasingly serious issue of antibiotic resistance, posing a significant threat to ecological safety and public health. Li et al. [94] developed a targeted responsive release Surface-Enhanced Raman Scattering (SERS) sensor (as shown in Figure 15A) based on a channel strategy of HP-UiO-66-NH_2_, which sensitively detects tetracycline (TTC). The hierarchical porous nanocontainer (HP-UiO-66-NH_2_ MOFs) features a large surface area and porous structure, effectively combining methylene blue (MB) and AuNP, with abundant metal active sites facilitating aptamer adsorption. The authors loaded MB and AuNP as signal probes and caps, respectively, into the synthesized metal–organic framework (HP-UiO-66-NH_2_ MOF). When tetracycline is present, the “molecular gate” of the aptamer opens, leading to the “release of cargo” from MB and AuNPs. Therefore, the concentration of TC can be determined by monitoring changes in the SERS intensity of the supernatant. Compared to other existing methods, the proposed targeted responsive release SERS sensor offers a wide detection range (0.01 to 10000 ng/mL) and a low LOD (0.01 ng/mL), achieving satisfactory recovery rates (93.23 to 108.79%) in milk and pork, demonstrating significant potential for this technology in antibiotic detection. Additionally, Lv et al. [95] developed a cascade amplification SERS aptasensor for the sensitive detection of tetracycline (TC) based on aptamers, enzyme-free DNA circuits, and SERS technology (Figure 15B). The DNA hairpins H1 and H2 were conjugated to the prepared Fe_3_O_4_@hollow-TiO_2_/Au nanonetworks (Fe_3_O_4_@h-TiO_2_/Au NC) and Au@4MBA@Ag nanoparticles, constructing capture and signal probes. Under optimal conditions, the aptasensor showed a significant linear response to TC, with a minimum detection limit of 15.91 pg·mL^−1^. Furthermore, the proposed cascade amplification sensing strategy exhibited good specificity and storage stability, and its practicality and reliability were validated by the detection of TC in real samples.

Meng et al. [96] proposed another SERS-based detection method for tetracycline, illustrated in Figure 15C. This method is based on Raman hotspots created between DNA sequence-coupled AuNPs (with diameters of 13 and 80 nm). The Raman signaling molecule (4MBA) is modified on the surface of the 13 nm AuNPs. Upon exposure to OTC, the aptamer sequence preferentially binds to OTC and unwinds its complementary strand, thereby bringing the 13 nm AuNPs closer to the 80 nm AuNPs. The stronger the interaction and generated hotspots, the higher the SERS signal. The SERS signal is positively correlated with OTC concentration, with a detection range from 9.27 × 10^−11^ to 9.27 × 10^−7^ μmol·L^−1^ and a detection limit as low as 8.77 × 10^−12^ μmol·L^−1^.

### 4.4. Application of Aptamer-Based SERS Technology in Biotoxin Detection

Biotoxins are toxic substances produced by various organisms (animals, plants, and microorganisms) and are considered natural toxins. Human experiences with biotoxins date back to food poisoning incidents. Long-term consumption of grains, meat, and vegetables contaminated with fungal toxins (especially from fermented foods) has remained a major cause of foodborne diseases [97]. With the deterioration of the environment and the improvement of living standards, there is increasing public concern about environmental pollution and food safety [98]. Biotoxins, as significant factors affecting both the environment and human health, have become more prominent [99]. Given their unique structures and the difficulty in finding antidotes, it is crucial to develop rapid, highly sensitive, and highly specific detection methods for biotoxins [100].

**Figure 16:**
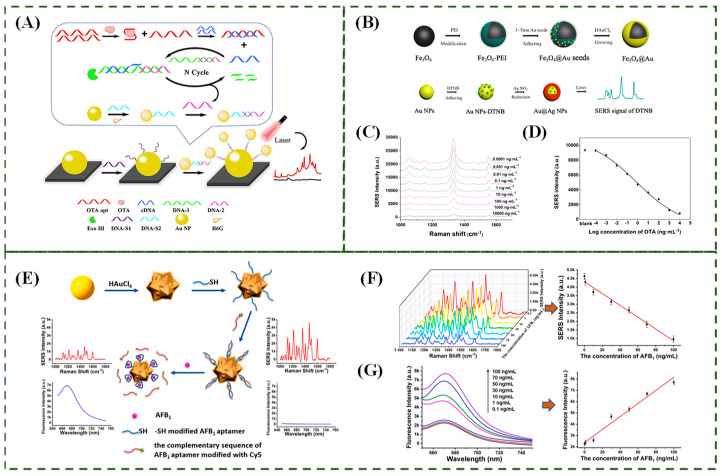
(**A**) Schematic diagram of the SERS sensor for the detection of OTA [101]. (**B**) Schematic diagram of the Fe_3_O_4_@Au magnetic nanoparticles (MGNPs) and Au-DTNB@Ag NPs [102]. (**C**) Typical Raman signal curves of OTA detection using the SERS-based aptasensor [102]. (**D**) Dose–response curve of OTA detection [102]. (**E**) Schematic illustration of the developed SERS/fluorescence dual mode nanosensor based on AuNFs for AFB1 detection. (**F**) The Raman and (**G**) fluorescence spectra of the nanosensor after incubation with different concentrations of AFB1. AuNFs/aptamer/DNA2 with different concentrations of DNA2. The corresponding calibration curve according to the SERS intensity at 1366 cm^−1^ and fluorescence intensity at 670 nm [103].

Huang et al. [101] developed a high-performance SERS biosensor (as shown in Figure 16A) for ultra-sensitive detection of biotoxins, based on a core-satellite combination and exonuclease-assisted dual amplification strategy. Using ochratoxin A (OTA) as an example, the applicability of the aptamer was tested. OTA is one of the most toxic and widely distributed biotoxins. The results showed that the limit of detection (LOD) for OTA reached 0.83 fg/mL. Furthermore, this dual amplification strategy has the potential to be applied to the detection of other DNA-specific targets, such as oligonucleotides, mi-RNA, and other DNA aptamer-targeted molecules. Aflatoxin M1 (AFM1) is a naturally occurring carcinogenic fungal toxin commonly found in milk. Song et al. [102] designed a SERS-based aptamer sensor for ochratoxin A (OTA), as shown in Figure 16B. Aptamer-GSNPs were used as SERS tags, while cDNA-MGNPs served as the signal-enhancing substrate and separation platform. An Au-DTNB@Ag-Fe_3_O_4_@Au complex was formed through oligonucleotide hybridization, where the Raman signal intensity of DTNB at 1331 cm^−1^ was significantly enhanced. As the concentration of OTA added to the complex increased, OTA preferentially bound to its aptamer, leading to the separation of the Raman signal probe (Aptamer-GSNPs) from the cDNA-MGNPs. This separation resulted in a lower Raman signal after magnetic separation. Based on this relationship, OTA can be quantitatively detected. Under optimal conditions, the detection limit of OTA was as low as 0.48 pg/mL (Figure 16B,C). Song et al. [103] developed a dual-mode nanosensor that combines the multi-optical signal detection capabilities of nanomaterials with the specific target recognition properties of aptamers. This sensor, illustrated in Figure 16E–G, utilizes gold nanoflowers (AuNFs) as the substrate. It was designed to detect Aflatoxin B1 (AFB1) with a detection limit of 0.03 ng/mL and a linear detection range spanning from 0.1 to 100 ng/mL.

**Figure 17:**
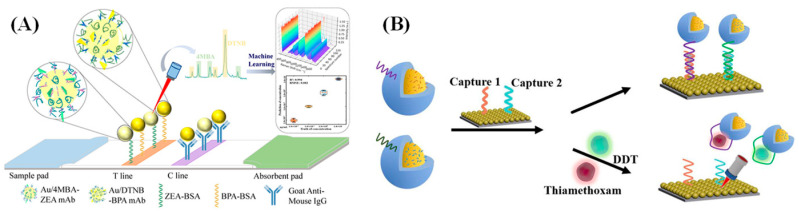
(**A**) Schematic diagram of qualitative to semiquantitative trace detection via SERS-ICA [104]. (**B**) Schematic diagram of multipesticide aptamer sensor detection principle [105].

As a rapid and nondestructive trace analysis technique, many research teams are committed to conducting broader explorations in the application fields of Surface-Enhanced Raman Scattering (SERS) technology. Our research group also focuses on this aspect and has carried out a series of in-depth studies aimed at issues such as the influence of the substrate signal’s nonuniformity on the SERS enhancement effect, the limitation of detection objects, and the interference from complex environments. For example, as shown in Figure 17A, Li et al. [104] combined the existing mature immunochromatographic assay (ICA) technology with SERS technology and introduced an internal standard method combined with deep learning to predict and process Raman data. On the basis of the signal fluctuations of single-antigen SERS-ICA test strips, this method constructed double-antigen SERS-ICA test strips. Full-spectrum Raman data from the double-antigen SERS-ICA test strips were normalized through two characteristic peaks of the internal standard molecule and then processed by a deep learning algorithm. After being processed using this method, the relative standard deviation (RSD) was improved by 3.8 times. After normalization, the prediction accuracy of the root mean square error (RMSE) improved 2.66 times and the prediction accuracy of the coefficient of determination (R^2^) increased from 0.961 to 0.994. Li et al. [105] established an aptamer-functionalized gold nanoparticle based on Surface-enhanced Raman spectroscopy (SERS) for the dual trace detection of harmful substances (Figure 17B). On the basis of the principle of aptamer complementary pairing, a “signal probe-target analyte-capture probe” structure was constructed to achieve the simultaneous indirect detection of thiamethoxam and dichlorodiphenyltrichloroethane (DDT). The results showed that the Raman signal intensities of thiamethoxam and DDT in the range of 10^−7^–10^−12^ M had a good linear relationship with the shifts of 4-MPY at 1582 cm^−1^ and 4-MBN at 1073 cm^−1^ (R_1582_ = 0.962; R_1073_ = 0.960). The detection limit reached 5.17 × 10^−1^³ M and 6.98 × 10^−1^³ M, respectively. This method has an excellent SERS enhancement effect and enhances the stability and reproducibility of SERS signals in the detection of pesticide residues in the field.

## 5. Conclusions and Perspective

Since its discovery, Surface-enhanced Raman scattering (SERS) has made significant progress in both scientific research and practical applications. Today, its applications have expanded to various fields, including chemical reaction analysis, biomarker detection, disease diagnosis, pollutant monitoring, and food safety testing, making it an important analytical tool. Especially in the bioassay field, SERS technology, with its high sensitivity and selectivity, has emerged as a promising biosensor technology. In recent years, SERS-based aptamer biosensors have gained widespread attention, and they are gradually becoming an important direction for SERS technology in small-molecule detection. This review discusses, in detail, the different types of aptamer-based SERS biosensors and their detection principles. We analyze their outstanding features, including sensitivity, specificity, and other performance indicators, while also exploring the advantages and limitations of these technologies. Aptamers, as novel molecular recognition elements, possess high affinity and specificity, enabling them to effectively recognize and detect target molecules in complex biological systems.

However, despite the extensive academic interest in aptamer-SERS technology, there are still some challenges, particularly in practical applications. For example, although numerous research studies have demonstrated the feasibility and superiority of this technology, there are still no commercially available products for widespread clinical or industrial use. To promote the large-scale application of this technology, future research should focus on developing handheld biosensor systems that can be mass-produced. These systems could overcome issues like high costs and complex operations inherent in traditional detection methods, offering significant market potential. However, achieving this goal still faces significant technical challenges. For instance, maintaining high sensitivity and good reproducibility across a large dynamic range remains a key problem in the development of SERS biosensor technology. Furthermore, the application of aptamer biosensors in live cells or in vivo is also an important direction. However, due to the complex biological environment, many Raman reporter molecules may interfere with biological molecules, thus affecting the accuracy of the signal. To address this issue, low-interference external reporter molecules (e.g., alkynes) could be used to avoid signal interference from biological molecules themselves. Additionally, because biological fluids contain various enzymes, many RNA aptamers are prone to degradation in biological media, which increases the difficulty of applying this technology. Therefore, improving the stability, durability, and environmental adaptability of aptamer biosensors is a critical challenge. Potential solutions include chemical modification of aptamer-SERS probes, such as surface modification with polyethylene glycol (PEG), to provide resistance to exonucleases. These measures can effectively extend the lifespan of aptamer-SERS probes and improve their stability in complex environments. To achieve more efficient detection, the development of high-throughput screening technology should also be promoted. By integrating multi-channel fluidic chips, parallel detection across multiple channels can be realized, accelerating the sample analysis process. Moreover, in the spectral analysis process, the use of multivariate analysis methods, such as chemometrics, can enhance the accuracy and reliability of data analysis. In conclusion, while aptamer-based SERS technology has demonstrated great potential in the field of biological detection, further breakthroughs in technology development and commercialization are necessary for its widespread application. By addressing key technical challenges such as stability, sensitivity, and reproducibility, and advancing high-throughput detection systems and intelligent analytical tools, aptamer-SERS biosensors have the potential to play an important role in clinical, industrial, and other practical applications. We believe that as research progresses, this technology will see broader applications in the future, driving technological advancements and innovation in related fields.
